# The ArcA regulon and oxidative stress resistance in *Haemophilus influenzae*

**DOI:** 10.1111/j.1365-2958.2007.05747.x

**Published:** 2007-06-01

**Authors:** Sandy M S Wong, Kishore R Alugupalli, Sanjay Ram, Brian J Akerley

**Affiliations:** 1 Department of Molecular Genetics and Microbiology, University of Massachusetts Medical School Worcester, MA 01655, USA.; 2 Division of Infectious Diseases and Immunology, University of Massachusetts Medical School Worcester, MA 01605, USA.

## Abstract

*Haemophilus influenzae* transits between niches within the human host that are predicted to differ in oxygen levels. The ArcAB two-component signal transduction system controls gene expression in response to respiratory conditions of growth and has been implicated in bacterial pathogenesis, yet the mechanism is not understood. We undertook a genome-scale study to identify genes of the *H. influenzae* ArcA regulon. Deletion of *arcA* resulted in increased anaerobic expression of genes of the respiratory chain and of *H. influenzae*'s partial tricarboxylic acid cycle, and decreased anaerobic expression levels of genes of polyamine metabolism, and iron sequestration. Deletion of *arcA* also conferred a susceptibility to transient exposure to hydrogen peroxide that was greater following anaerobic growth than after aerobic growth. Array data revealed that the *dps* gene, not previously assigned to the ArcA modulon in bacteria, exhibited decreased expression in the *arcA* mutant. Deletion of *dps* resulted in hydrogen peroxide sensitivity and complementation restored resistance, providing insight into the previously uncharacterized mechanism of *arcA*-mediated H_2_O_2_ resistance. The results indicate a role for *H. influenzae arcA* and *dps* in pre-emptive defence against transitions from growth in low oxygen environments to aerobic exposure to hydrogen peroxide, an antibacterial oxidant produced by phagocytes during infection.

## Introduction

*Haemophilus influenzae* has no identified natural niche outside of the human host where it primarily colonizes the nasopharyngeal mucosa. It can disseminate to other anatomical sites making it a common cause of otitis media, upper and lower respiratory tract infections, septicaemia and meningitis in children (Klein, 1997; Moxon and Murphy, 2000). *H. influenzae* also frequently colonizes the respiratory tract of individuals with chronic obstructive pulmonary disease (Sethi and Murphy, 2001; Murphy and Sethi, 2002; Murphy *et al*., 2004) and cystic fibrosis (Gilligan, 1991; Moller *et al*., 1995). The incidence of *H. influenzae* meningitis has dramatically declined in populations immunized with an effective vaccine against the type b capsular polysaccharide [Centers for Disease Control and Prevetion (CDC), 2002], a major factor promoting bloodstream survival by strains of this serotype. However, the vaccine has not affected the incidence of infection with non-typeable strains (NTHi), which lack the capsule. Although NTHi predominantly cause respiratory tract infections and otitis media, they have been isolated from patients with invasive disease such as meningitis in rare cases, raising the possibility that genes conferring varying degrees of bloodstream persistence could be distributed among NTHi strains (Nizet *et al*., 1996; Cuthill *et al*., 1999; O'Neill *et al*., 2003; Erwin *et al*., 2005).

We postulate that modulation of gene expression in response to environmental conditions is required by *H. influenzae* to express the repertoire of genes needed for survival during pathogenesis. *H. influenzae* likely encounters varying oxygen levels in diverse environments in the host such as growth in biofilm structures on mucosal surfaces or after invasion into the bloodstream. Signal transduction in response to varied oxygen levels represents a mechanism by which *H. influenzae* could co-ordinate gene expression profiles needed for efficient colonization and pathogenesis in different environments encountered within the host. In *Escherichia coli*, a two-component signal transduction system designated ArcAB (for anoxic redox control) responds to respiratory conditions of growth to modulate expression of genes/operons of the tricarboxylic acid (TCA) cycle and genes involved in other aspects of respiratory or fermentative metabolism (Lynch and Lin, 1996a). The ArcB sensor kinase autophosphorylates and transfers a phosphoryl group to ArcA, a DNA binding protein that can act as either repressor or activator depending on the configuration of the target promoter (Lynch and Lin, 1996a,b). The ArcAB system is most active under low oxygen conditions and least active under high oxygen conditions. Recent evidence indicates that this response is likely not via direct sensing of oxygen but that ArcB senses the oxidation or reduction (redox) status of the membrane-bound quinones, central electron carriers of respiration (Georgellis *et al*., 2001a; Malpica *et al*., 2004). The ArcAB system of *H. influenzae* possesses similar biochemical and regulatory functions to those of its counterpart in *E. coli* in modulating gene expression in response to redox conditions of growth. Of note, expression of *H. influenzae* ArcB in an *E. coli arcB* mutant can restore the response of at least two ArcAB-regulated promoters, *sdh* (succinate dehydrogenase) and *lldP* (l-lactate permease), to respiratory conditions of growth (Manukhov *et al*., 2000; Georgellis *et al*., 2001b). Several genes or proteins that are repressed by ArcA in *E. coli* have been identified as ArcA-regulated in *H. influenzae*, including *lldD* (l-lactate dehydrogenase) and certain subunits of formate dehydrogenase and fumarate reductase (Georgellis *et al*., 2001b; De Souza-Hart *et al*., 2003). ArcA is a global regulator in *E. coli*, however, the extent of the ArcA regulon of *H. influenzae* is unknown.

ArcA has been implicated in pathogenesis as *arcA* mutants of both *H. influenzae* and *Vibrio cholerae*, a diarrhoeal pathogen, exhibit reduced lethality compared with wild type in mouse mortality studies (De Souza-Hart *et al*., 2003; Sengupta *et al*., 2003). Despite extensive information concerning ArcA-mediated control of genes of respiratory pathways and enzymes of the TCA cycle in *E. coli*, the mechanism by which this gene regulation could alter virulence in *H. influenzae* is not well understood. ArcA mutants of *H. influenzae* type b were more sensitive than wild type to killing by human serum, however, ArcA-regulated genes encoding cell-surface structures as potential targets of humoral immune components in serum, such as complement, have yet to be identified. *V. cholerae* ArcA influences production of cholera toxin which is essential for virulence, yet *H. influenzae* produces no exotoxins implicated in pathogenesis. In *Salmonella enterica* serovar Enteritidis, ArcA has been implicated in resistance to reactive oxygen and nitrogen intermediates (ROI/RNI) (Lu *et al*., 2002). A role in oxidative stress resistance for a regulator such as ArcA, which is active under low oxygen conditions, appears to be paradoxical, and the mechanism and role of ArcA-regulated genes in this resistance profile has not been determined.

In the current study, we have extended our analysis of the *H. influenzae* ArcAB system to understand mechanisms by which this signalling system can influence *H. influenzae* pathogenesis. We analysed the global expression profile of the *H. influenzae arcA* mutant grown under anaerobic conditions to identify genes comprising the ArcA regulon in this organism. By microarray analysis, we identified a set of genes whose expression pattern was influenced by the *arcA* mutation and restored by complementation. Northern hybridizations confirmed ArcA-mediated control of all of the genes that were evaluated by this method. In addition to detecting genes encoding respiratory metabolic enzymes known to be ArcA-regulated in *E. coli,* this analysis identified and validated ArcA-dependent modulation of genes not previously recognized to be within the ArcA regulon. One of these genes is a putative homologue of Dps proteins in other species that participate in oxidative stress resistance yet have not been previously linked to ArcA-mediated phenotypes. Thus, the microarray results gave us insight into physiological characteristics of the *H. influenzae arcA* mutant that can account for its oxidative stress sensitivity. Mutational analysis of ArcA controlled genes including *dps* provided insight into the mechanism of ArcA-mediated resistance to hydrogen peroxide, and provides support for a model in which ArcA promotes survival of cells shifted from low oxygen conditions to oxidative stress exposure, a transition *H. influenzae* is likely to experience in the host.

## Results

### *Global expression profiling of the* H. influenzae arcA *mutant*

To investigate the role of the ArcAB signal transduction system in *H. influenzae* pathogenesis, the ArcA regulon in *H. influenzae* Rd was investigated by DNA microarray analysis. Genomic expression profiles were measured in four independent samples each of the parent strain, RdV, the *arcA* deletion mutant, RAA6V (Δ*arcA*), and the complemented strain, RAA6C (Δ*arcA*, *arcA*^+^), grown anaerobically. The complete set of data from these experiments is provided in the web supplement (Table S1 and S2). Of the 1697 *H. influenzae* protein coding genes represented on the array, expression of 19 genes was increased by greater than or equal to twofold (*P*-value ≤ 0.0001) in the Δ*arcA* mutant, RAA6V, compared with its parent, RdV (Table 1, RAA6V/RdV column). Expression of these genes was restored to levels similar to that of RdV in the complemented strain, RAA6C (Table 1, RAA6V/RAA6C column) providing a high degree of confidence in this set of candidate ArcA modulated genes. Six genes that showed increased expression in the Δ*arcA* mutant include genes encoding putative homologues of *E. coli* dehydrogenases of the respiratory chain (*fdxH*, *fdxI*, *ndh* and *lldD*) or the TCA cycle (*sucA* and *sucB*). The Δ*arcA* mutant also exhibited increased expression of a putative homologue of *lipA*, which is involved in the biosynthesis of lipoate, a cofactor used by several enzyme complexes involved in oxidative metabolism such as pyruvate dehydrogenase and α-ketoglutarate dehydrogenase (Vanden Boom *et al*., 1991). Two other genes whose expression was negatively controlled by ArcA are similar to *E. coli* genes, *lldP* and *fdhE,* that encode lactate permease and a protein involved in assembly of formate dehydrogenase, respectively (Dong *et al*., 1993; Abaibou *et al*., 1995; Nunez *et al*., 2002). Expression levels of five genes that were decreased in the Δ*arcA* mutant compared with its parent, RdV, were restored in the complemented strain, generating a list of ‘high confidence’ candidate *arcA* activated genes (Table 2). One of the five genes was *arcA* itself as expected because the gene was deleted; three are genomically linked in organization, *HI0590* (*potE*), *HI0591* (*speF*) and *HI0592*. The fifth gene, *HI1349*, encodes a predicted protein with sequence similarity to Dps proteins that function in protection against oxidative stress (Almiron *et al*., 1992; Ilari *et al*., 2000; Pulliainen *et al*., 2005).

**Table 1:** Genes increased in expression in the *H. influenzae arcA* deletion mutant compared with parent strain.

		RAA6V/RdV		RAA6V/RAA6C	
			
Gene ID	Function	Fold change	*P*-value	Fold change	*P*-value
HI1218	l-lactate permease (*lldP*)	44	1.20E-08	26.8	1.69E-08
HI0747	NADH dehydrogenase (*ndh*)	11	4.06E-11	9.6	5.86E-11
HI0009	FdhE protein (*fdhE*)	9.7	8.39E-10	9.4	1.53E-09
HI0008	Formate dehydrogenase, gamma subunit (*fdxI*)	9.7	5.42E-09	12	3.81E-09
HI0007	Formate dehydrogenase, beta subunit (*fdxH*)	7.8	1.12E-09	9.2	7.77E-10
HI1731	Conserved hypothetical protein	5.9	2.52E-09	6.2	4.67E-09
HI1444	5,10 methylenetetrahydrofolate reductase (*metF*)	5.6	4.91E-06	4.1	2.57E-05
HI0608	Conserved hypothetical protein	5.5	3.65E-10	8.3	1.81E-10
HI1727	Argininosuccinate synthetase (*argG*)	4.8	5.02E-07	4.8	3.56E-07
HI1728	Conserved hypothetical protein	3.5	1.54E-06	3.1	2.58E-05
HI1739.1	l-lactate dehydrogenase (*lldD*)	3.2	1.79E-10	4.5	1.07E-11
HI1730	Conserved hypothetical protein	3.0	1.48E-06	7.0	3.32E-08
HI1661	2-oxoglutarate dehydrogenase E2 component (*sucB*)	2.8	5.33E-07	3.0	1.14E-06
HI0026	Lipoate biosynthesis protein A (*lipA*)	2.6	4.08E-07	2.2	9.63E-06
HI1662	2-oxoglutarate dehydrogenase E1 component (*sucA*)	2.6	6.71E-07	3.5	1.07E-07
HI0018	Uracil DNA glycosylase (*ung*)	2.5	1.01E-05	2.2	5.68E-05
HI0890	Dephospho-CoA kinase (*coaE*)	2.5	4.83E-05	2.4	2.91E-04
HI0889	Serine hydroxymethyltransferase (serine methylase) (*glyA*)	2.4	4.11E-06	2.5	6.13E-06
HI0174	Conserved hypothetical protein	2.3	1.75E-05	2.1	1.92E-04

List contains genes whose expression levels were increased in the Δ*arcA* mutant, RAA6V compared with the parent strain, RdV (column RAA6V/RdV) and were restored close to parental levels in the complemented strain, RAA6C (column RAA6V/RAA6C). Fold differences are ≥ 2.0 with *P* ≤ 0.0001.

**Table 2:** Genes decreased in expression in the *H. influenzae arcA* deletion mutant compared with parent strain.

		RAA6V/RdV		RAA6V/RAA6C	
			
Gene ID	Function	Fold change	*P*-value	Fold change	*P*-value
HI0884	Aerobic respiration control protein ArcA (*arcA*)	−654.8	2.72E-12	−776.7	9.02E-10
HI0592	Conserved hypothetical protein	−4.4	4.62E-08	−3.8	6.36E-05
HI0591	Ornithine decarboxylase (*speF*)	−4.0	2.54E-08	−2.7	2.49E-05
HI1349	Conserved hypothetical protein, similar to *dps* protein family	−3.4	1.43E-07	−3.0	5.97E-06
HI0590	Putrescine-ornithine antiporter (*potE*)	−2.5	3.06E-08	−2.8	5.27E-07

List contains genes whose expression levels were decreased in the Δ*arcA* mutant, RAA6V compared with the parent strain, RdV (column RAA6V/RdV) and were restored in the complemented strain, RAA6C (column RAA6V/RAA6C). Fold differences are ≥ 2.0 with *P* ≤ 0.0001.

**Fig. 1:**
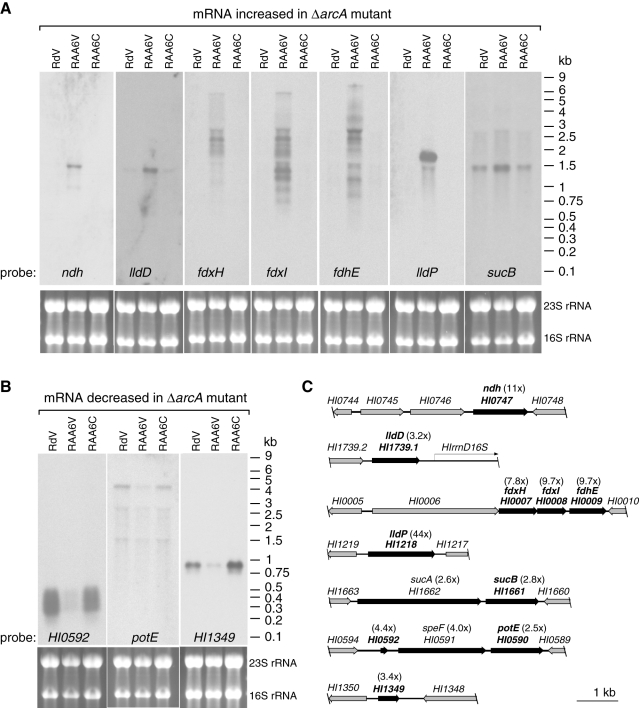
Differential transcript abundance in the *H. influenzae arcA* mutant versus parental strain. Northern blots containing 6 μg of total RNA from anaerobically grown Rd parental strain, RdV, *arcA* deletion mutant, RAA6V (Δ*arcA*) and complemented strain, RAA6C hybridized with probes corresponding to genes increased (A) or decreased (B) in expression in the Δ*arcA* mutant (Tables 1 and 2 respectively). Ethidium bromide stained gel is directly below each blot. 16S and 23S are the ribosomal RNAs. C. Schematic representation of the genomic organization of genes modulated in the Δ*arcA* mutant (black open reading frames) of which the genes in bold were evaluated on Northern blots in panels A and B. Numbers in parentheses near each gene represent the fold change in differential gene expression in the Δ*arcA* mutant versus parent comparison (Tables 1 and 2).

To verify the microarray results, we performed Northern blot hybridizations with genes whose expression as detected on the arrays either increased in the Δ*arcA* mutant (*ndh*, *lldD*, *fdxH*, *fdxI*, *fdhE*, *lldP* and *sucB*) or decreased in the Δ*arcA* mutant (*HI0592*, *potE* and *HI1349*) (Tables 1 and 2; Fig. 1). Northern blots containing RNA from the parent strain, RdV, the Δ*arcA* mutant, RAA6V, and the complemented strain, RAA6C, grown anaerobically were analysed with probes specific to these genes (*Experimental procedures*). Levels of each of the *ndh*, *lldD*, *fdxH*, *fdxI*, *fdhE*, *lldP* and *sucB* specific transcripts were higher in the Δ*arcA* mutant compared with its parent and complementation restored negative control of these genes (Fig. 1A and C). Levels of the *HI0592*, *potE* and *HI1349* specific transcripts were decreased in the Δ*arcA* mutant compared with its parent and transcript abundance was restored with complementation (Fig. 1B). Transcript sizes for each of the loci appear to be generally consistent with the gene lengths annotated by The Institute for Genomic Research (TIGR) (Fig. 1C). The mRNAs of multiple sizes that were detected with the *fdxH*, *fdxI* and *fdhE* specific probes are likely to represent polycistronic transcripts with the largest ∼3 kb transcript potentially spanning *fdxH*, *fdxI* and *fdhE*. The large mRNA species (∼4 kb) detected with the *potE* probe is likely to represent a polycistronic transcript spanning *speF-potE* or *HI0592-speF-potE*. Based on our subsequent results implicating *HI1349* in ArcA-mediated phenotypes (see below), this gene's expression pattern was additionally verified by reverse transcription quantitative polymerase chain reaction (RT-qPCR) and found to be decreased by 4.6-fold in the Δ*arcA* mutant compared with its parent and by 2.7-fold in comparison to the complemented strain. These results are in agreement with the microarray data and confirm ArcA-dependent modulation of these genes in *H. influenzae*.

### *Sensitivity of the* H. influenzae arcA *mutant to oxidative stress*

The microarray data suggested that the *H. influenzae*Δ*arcA* mutant might exhibit sensitivity to oxidative stress, a phenotype of relevance to the role of *arcA* in bacterial survival in the host. Our expression analysis detected putative homologues of eight genes encoding subunits or assembly factors of respiratory chain dehydrogenases (*fdxH*, *fdxI*, *fdxE*, *ndh* and *lldD*), a dehydrogenase substrate transporter (*lldP*), or TCA cycle enzymes (*sucA* and *sucB*) that were increased in expression in the *H. influenzae*Δ*arcA* mutant. An increase in respiratory activity mediated by these genes in the *H. influenzae*Δ*arcA* mutant could produce elevated levels of ROI during a transition from anaerobiosis to an oxygenated microenvironment (see *Discussion*).

Conversely, the candidate ArcA activated genes likely promote resistance to oxidative stress. Two genes whose expression levels were lower in the *arcA* mutant have potential roles, based on sequence similarity to their *E. coli* counterparts, in the biosynthesis of the polyamine putrescine (*speF*), or transport of putrescine (*potE*) (Table 2). The putative *H. influenzae* SpeF and PotE show 65% and 77% amino acid identity to *E. coli* SpeF and PotE respectively. Polyamines are present in all organisms and are associated with a variety of vital biological processes such as replication, transcription and cell growth (Pegg, 1988; Tabor and Tabor, 1985). Addition of exogenous polyamines can protect polyamine deficient *E. coli* from the toxicity of the reactive oxygen species H_2_O_2_ (Tkachenko *et al*., 2001; Chattopadhyay *et al*., 2003; Jung and Kim, 2003). Moreover, *HI1349*, encoding a Dps-like protein, was also downregulated in the Δ*arcA* mutant (Table 2). HI1349 has approximately 18% identity and 20% similarity to *E. coli* Dps and contains a conserved iron-binding motif found in Dps homologues (see *Discussion*). Members of the ferritin-like Dps protein family function in iron storage/detoxification (Chiancone *et al*., 2004). Dps was first discovered in *E. coli* as a DNA-binding protein that protects DNA from hydrogen peroxide-mediated oxidative damage (Almiron *et al*., 1992). Nearly half (11/23) of the detected ArcA controlled genes in *H. influenzae* have potential roles in ROI generation or resistance to oxidative stress. Therefore, in addition to its apparent role in optimizing metabolic flux under anaerobic conditions, it is possible that ArcA plays a role in pre-emptive protection against exposure of anaerobically grown cells to oxidants. Potentially consistent with this hypothesis, the ArcA of *S. enterica* serovar Enteritidis has been implicated in resistance to reactive oxygen and nitrogen intermediates by an unknown mechanism (Lu *et al*., 2002).

**Fig. 2:**
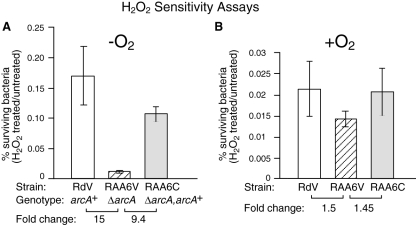
Sensitivity of the *H. influenzae arcA* mutant to hydrogen peroxide. The parental strain, RdV, *arcA* deletion mutant, RAA6V (Δ*arcA*) and complemented strain, RAA6C (Δ*arcA, arcA*+) from anaerobically (A) and aerobically (B) grown cultures were treated with 0.5 mM H_2_O_2_ for 10 min (*Experimental procedures*). The survival ratios are plotted as the percentage of cfu obtained from H_2_O_2_ treated/untreated samples. Values represent the mean of three independent cultures of each strain tested, and the error bars represent the standard deviations. Statistically significant differences between the *arcA* mutant and parent (*P* < 0.01) and between the *arcA* mutant and complemented strain (*P* < 0.05) were observed (one-way anova with Bonferroni's multiple comparison test) for cultures grown anaerobically. The *arcA* mutant exhibits a 15-fold and 1.5-fold increase in sensitivity to H_2_O_2_ challenge compared with parent when grown anaerobically and aerobically respectively.

To determine if *arcA* has a role in protection of *H. influenzae* during a transition from anaerobic growth to aerobic oxidative stress conditions, we tested the effect of transient H_2_O_2_ exposure on the viability of the *H. influenzae*Δ*arcA* mutant grown under anaerobic versus aerobic conditions. We could detect no appreciable differences in growth rates of the Δ*arcA* mutant, the parental strain and the complemented strain under these conditions alone (*Experimental procedures*). Anaerobic or aerobic cultures of the parent strain, RdV, the Δ*arcA* mutant and the complemented strain were incubated aerobically for 10 min in the absence and presence of 0.5 mM H_2_O_2_ followed by quenching of the H_2_O_2_ with sodium pyruvate before plating to enumerate survivors (Fig. 2). Consistent with greater activity of ArcA under low oxygen conditions, anaerobically grown Δ*arcA* mutant exhibited an approximate 15-fold decrease in the number of survivors compared with its parent after H_2_O_2_ treatment and complementation restored the survival phenotype to near the parental level (Fig. 2A). Similar fold differences in survival were obtained with exposure of anaerobic cultures to 0.25 mM H_2_O_2_ (10.5 ± 0.9% survival of the parental strain, 0.5 ± 0.3% survival of the Δ*arcA* mutant, and 10.1 ± 1.1% survival of the complemented strain). Growth in an anaerobic chamber prior to H_2_O_2_ exposure at either concentration yielded similar fold differences in survival compared with the sealed tube condition (data not shown). In contrast, the aerobically grown Δ*arcA* mutant showed only a 1.5-fold decrease relative to the parental strain in the number of survivors after exposure to H_2_O_2_ and complementation restored survival similar to the parental level (Fig. 2B).

To evaluate whether the *speF*, *potE* and *HI0592* genes participate in the H_2_O_2_ sensitivity phenotype as observed in the *arcA* mutant, we created a strain, RputV, which contains a deletion of all three loci. Anaerobic growth of RputV, followed by challenge with 0.5 mM H_2_O_2_ resulted in a slight reduction (∼14%) in the number of survivors in the mutant compared with the parental strain that did not reach statistical significance (data not shown).

**Fig. 3:**
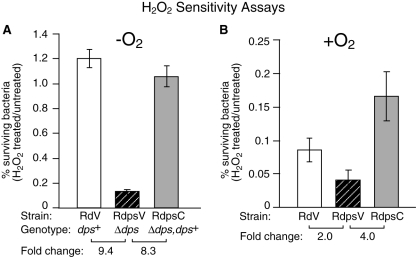
Sensitivity of the *H. influenzae HI1349* (*dps*) mutant to hydrogen peroxide. H_2_O_2_ sensitivity assays with the parental strain, RdV, *dps* deletion mutant, RdpsV (Δ*dps*) and complemented strain, RdpsC (Δ*dps, dps*+), from anaerobically (A) and aerobically (B) grown cultures were performed as described in Fig. 2. Statistical differences: parent versus *dps* mutant (*P* < 0.001 for anaerobic growth, *P* > 0.05 for aerobic growth); complemented strain versus *dps* mutant (*P* < 0.001 for anaerobic and aerobic growth) (one-way anova with Bonferroni's multiple comparison test). The *dps* mutant exhibits a 9.4-fold and twofold increase in sensitivity to H_2_O_2_ challenge compared with parent when grown anaerobically and aerobically respectively.

Next, we examined the role of the putative *dps* homologue, *HI1349* in resistance to H_2_O_2_. We observed that deletion of this locus renders the mutant, RdpsV, more sensitive to H_2_O_2_ challenge compared with the parent when grown anaerobically, as it showed approximately ninefold reduction in the number of survivors (Fig. 3A). In contrast, H_2_O_2_ treatment of the aerobically grown RdpsV resulted in only a twofold decrease in the number of survivors compared with the parent, RdV (Fig. 3B). Under either condition, complementation restored the survival phenotypes of the mutant to levels at or above those of the parental strain. Based on sequence and motif similarity to putative homologues in other species, together with its role in H_2_O_2_ resistance demonstrated here, *HI1349* is referred to as *dps* in this report.

These results indicate that the *H. influenzae arcA* mutant has an increased sensitivity to H_2_O_2_ following anaerobic growth. In addition, *dps*, a gene identified as a target of ArcA control in these experiments, plays a role in resistance to H_2_O_2_ hypersensitivity providing evidence for a mechanism by which ArcA mediates protection from ROI in this species.

### *Effect of arcA on survival of* H. influenzae *in a murine model of bacteraemia*

Intravascular colonization is a well-established virulence trait of *H. influenzae*. Bacteraemia is primarily associated with encapsulated strains, which are of declining clinical significance in countries with adequate Hib vaccination programs. Nevertheless, recent evidence indicates that a subset of NTHi strains, which are non-encapsulated, also has the capacity to infect the bloodstream (Nizet *et al*., 1996; Cuthill *et al*., 1999; O'Neill *et al*., 2003). Bloodstream colonization by NTHi is less efficient and persistent than is observed with encapsulated strains, yet it is possible that a limited ability to infect the mammalian bloodstream and resist immunological clearance is a general feature of *H. influenzae* that varies quantitatively between diverse isolates. For these studies we used a nonencapsulated Rd strain clonally related to KW20, whose genome sequence has been available for many years and has provided a reference point for genetic analysis of *H. influenzae* (Fleischmann *et al*., 1995). Rd strains are efficiently transformable, unlike typical NTHi clinical isolates. Despite exhibiting less virulence than encapsulated strains, Rd derivatives generate transient infections in animal models of infection and have been useful for characterization of pathogenic properties of *H. influenzae* (Weiser *et al*., 1995; Daines *et al*., 2003). The recently determined genome sequence of NTHi strain 86–028NP contains putative homologues of ∼97% of Rd genes indicating that genes implicated in infection related phenotypes with Rd are potentially present in other *H. influenzae* strains, although some unique genes present in clinical NTHi isolates are absent from Rd (Erwin *et al*., 2005; Harrison *et al*., 2005).

**Fig. 4:**
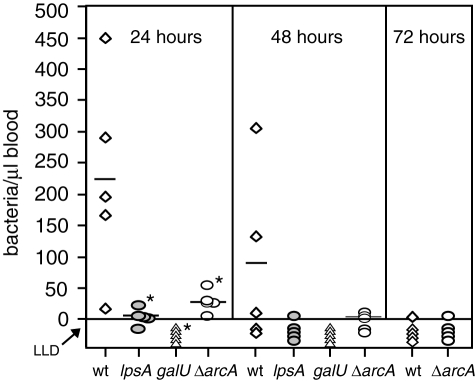
Persistence defect of *H. influenzae* mutants in a mouse model of bacteraemia. *H. influenzae* Rd wild-type (wt), Rcp5 (*lpsA*) and Rcp19 (*galU*), and *arcA* deletion mutant (Δ*arcA*) were inoculated IP into five C57BL/6 mice per strain and blood was sampled daily for cfu determination (*Experimental procedures*). Bars represent the average cfu μl^−1^ (224 and 28 for wt and Δ*arcA* mutant, respectively, at 24 h post inoculation). Asterisks denote differences from wild type that were statistically significant (*P* < 0.01). lower limit of detection (LLD) = 2 cfu μl^−1^.

As expected for non-encapsulated *H. influenzae*, Rd produced a transient infection in mice. Bacteria were recovered at an average density of 224 colony-forming units (cfu) μl^−1^ of blood at 24 h after intraperitoneal (IP) inoculation, began to decline by 48 h, and were fully cleared by 72 h post inoculation (Fig. 4). The ability of this model to detect *H. influenzae* virulence properties is demonstrated by comparison of the Rd strain and isogenic mutants deficient in *lpsA* or *galU,* lipooligosaccharide (LOS) biosynthesis genes essential for bloodstream colonization by *H. influenzae* type b in an infant rat model (Hood *et al*., 1996). The *lpsA* gene is the glycosyltransferase required for glucosyl addition and extension of the third heptosyl residue of the LOS inner core, and *galU* encodes the UDP-glucose pyrophosphorylase required for production of the UDP-glucose precursor essential for all hexose additions to the LOS. No viable bacteria were recovered at any time point from mice infected with the *galU* mutant. Likewise, mutation of *lpsA* yielded a similar level of attenuation to that of the *galU* mutation. Therefore, this model is capable of detecting predicted phenotypes associated with the classical virulence factors of *H. influenzae*.

If signalling in response to redox conditions is important for adaptation to niches encountered by *H. influenzae* during infection, then *arcA* may be expected to play a role in some aspect of survival or persistence in the host. A mutant containing a non-polar deletion of the *arcA* gene was detected at an average density of 28 cfu μl^−1^ of blood at 24 h post inoculation. This represents an approximately eightfold reduction in cfu relative to the parent strain at 24 h post inoculation (Fig. 4). At 48 h, the Δ*arcA* mutant remains attenuated, although the difference at this later time did not reach statistical significance. Consistent with these results, De Souza-Hart *et al*. have shown that an *arcA* mutation confers an increase in LD50 with *H. influenzae* type b after IP inoculation of BALB/c mice (De Souza-Hart *et al*., 2003). This virulence defect was attributed to the increased serum sensitivity of the *arcA* mutant that was observed. We evaluated the Rd Δ*arcA* mutant versus parental strain for serum sensitivity after anaerobic growth. Both strains were sensitive to treatment with human serum with an approximate IC50 of 2% (*Experimental procedures*) and did not differ detectably in serum sensitivity in three independent experiments: survival ratio in 2% serum: Rd (parent) = 0.57, standard deviation (SD) = 0.1; RAA6 ( Δ*arcA*) = 0.56, SD = 0.04. It therefore seems likely that the role of ArcA in serum resistance differs between strains. Nevertheless, the more rapid clearance of the *arcA* mutant from the blood raises the possibility that an impaired ability to maintain high numbers of bacteria in the blood contributes to the previously observed reduction in lethality of the *arcA* mutant versus the parental type b strain. Furthermore, decreased ability of the mutant to survive in the mammalian bloodstream suggests that ArcA is active in controlling genes during a critical stage of pathogenesis.

Because deletion of the ArcA-regulated *dps* gene resulted in an increased sensitivity to H_2_O_2_ (Fig. 3), we evaluated whether this gene could contribute to *H. influenzae's* ability to persist in the host bloodstream. We coinoculated 6-week-old C57BL/6 mice by the IP route at a dose of 2 × 10^8^ cfu of *H. influenzae* Rd carrying the *lacZ* gene (strain RdlacZ) and an equal number of either the parent (strain RdV), Δ*dps* mutant (RdpsV), or complemented *dps* strain (RdpsC). After 24 h, the ratios and standard deviations of the coinoculated strains recovered from the blood were not significantly different: RdV/RdlacZ = 1.32, SD = 0.73, *n* = 4; RdpsV/RdlacZ = 2.27, SD = 0.74, *n* = 4; RdpsC/RdlacZ = 0.97, SD = 0.22, *n* = 5. The results indicate that the Δ*dps* mutant does not appear to exhibit a survival defect compared with the parental strain in the bloodstream at 24 h post inoculation. The *H. influenzae* ArcA regulates multiple genes as indicated by expression profiling and it is likely that the combined effect of more than one gene expressed at inappropriately high or low levels in the ArcA mutant confers the *in vivo* defect.

## Discussion

### Overview

We report the role of *H. influenzae* ArcA in resistance to hydrogen peroxide during transitions between redox conditions of growth and enhanced survival in a murine model of bloodstream clearance. Application of DNA microarray-based expression profiling led to the identification of an ArcA controlled gene, *dps* which encodes a putative homologue of the ferritin-like Dps protein and has not previously recognized to be within bacterial ArcA regulons. The *H. influenzae dps* gene was implicated in ArcA-mediated resistance to hydrogen peroxide, providing evidence for a molecular mechanism of ArcA-mediated ROI resistance. It will be of interest to determine whether ArcA controls genes fulfilling similar functions in other species, or whether ArcA-mediated regulation of *dps* is unique to this inhabitant of the human respiratory tract.

Of the 1697 protein coding genes represented on the microarray, we found 23 (∼1.3% of the 1740 genes in the genome) that were differentially expressed in the *arcA* mutant when compared with its parent strain and complemented *arcA* strain (Tables 1 and 2). The *H. influenzae arcA* mutant exhibited increased anaerobic expression of genes of the respiratory chain and TCA cycle, and decreased anaerobic expression levels of genes with putative functions in polyamine biosynthesis/transport and oxidative stress resistance. In this mutant, a potential increase in cellular respiration could lead to an increase in intracellular generation of ROI, whereas a concurrent decrease in the uptake/biosynthesis of polyamines and in the levels of the putative Dps protein could lower resistance to ROI.

This model based on the expression data suggested a mechanism by which the *H. influenzae* ArcA could promote oxidative stress resistance. We found that mutants containing non-polar deletions of either the *H. influenzae arcA* or *HI1349* (a putative *dps* homologue), a gene shown in this study to be positively regulated by *arcA,* possess enhanced sensitivity to H_2_O_2_ following a shift from growth in low oxygen to a transient aerobic exposure to this oxidant. We postulate that as *H. influenzae* transits from low to high oxygen environments within the host, ArcAB protects the cell against oxidative stress, contributing to the ability of *H. influenzae* Rd to resist clearance in a mouse model of intravascular infection as we report here.

Resistance to oxidative stress is a major feature of bacterial pathogens that must survive encounters with defences of the host innate immune system (Nathan and Shiloh, 2000). The importance of this trait in *H. influenzae* is supported by the observation that the *sodA* gene encoding superoxide dismutase, which was not detected by our microarray analysis as an ArcA-regulated gene in *H. influenzae*, is required for oxidative stress defence *in vitro* and for optimal nasopharyngeal colonization in infant rats (D'Mello *et al*., 1997). Our results suggest that co-ordinate regulation of additional oxidative stress resistance pathways could represent a mechanism by which ArcA promotes survival of *H. influenzae* during bloodstream infection.

### ArcA regulon diversity

Compared with the relatively small number of ArcA targets in *H. influenzae* identified in the microarray data, global expression profiling experiments conducted with *E. coli arcA* mutants have indicated that transcript levels of 372 genes (Liu and De Wulf, 2004) or as many as 1139 genes (Salmon *et al*., 2005) (∼9% and ∼26%, respectively, of the *E. coli* genome) are controlled directly or indirectly by ArcA anaerobically, while 110 genes were found to be ArcA-regulated under an aerobic growth condition (Oshima *et al*., 2002). Use of an ArcA-P recognition weight matrix from footprinting data for 10 known ArcA-regulated genes identified approximately 50 additional *E. coli* operons as probable direct targets of ArcA (Liu and De Wulf, 2004). The difference in the number of ArcA targets between *H. influenzae* and *E. coli* is likely related, in part, to their difference in genome size (the *E. coli* genome is ∼2.3-fold larger) and host environment. *H. influenzae* inhabits a more restricted environment (primarily the human nasopharynx) compared with *E. coli*. In addition, some ArcA-regulated promoters in *E. coli* are subject to complex combinatorial control by other regulators such as FNR, which is considered a direct sensor of oxygen and is active under a more restricted range of low oxygen conditions than ArcA (Lynch and Lin, 1996a; Kiley and Beinert, 1998). It is likely that under diverse environmental conditions, different subsets of ArcA-regulated genes may be detected as a result of the presence or absence of contributions from other regulatory systems.

Of the 23 ArcA modulated genes on our list (Tables 1 and 2, columns RAA6V/RdV), all but *HI0592* have putative homologues in *E. coli* with 10 showing agreement in anaerobic expression ratios in *arcA* versus parental strains in the two species suggesting similarities and differences in their respective *arcA* regulons (Liu and De Wulf, 2004; Salmon *et al*., 2005) (*Supplementary material*, Table S3). These results are consistent with regulon diversity among putative ArcA homologues in other species. Of note, Gralnick and coworkers (Gralnick *et al*., 2005) applied the *E. coli* ArcA-P recognition weight matrix to the genome of *Shewanella oneidensis* and experimentally validated several candidates detected by this method. In *S. oneidensis*, the *dmsEFAB* genes, encoding dimethyl sulphoxide reductase, were positively controlled by ArcA and the *cydAB* genes, encoding cytochrome *d* oxidase, were negatively controlled. In contrast, *dms* genes in *E. coli* are not known to be ArcA-regulated and *cydAB* of *E. coli* is ArcA activated (Lynch and Lin, 1996b). Each of these three bacterial species inhabits distinctly different environments in nature and it is likely that their respective ArcA regulons are each uniquely adapted to growth and survival in these settings.

### *Negative control of genes of respiration and the TCA cycle by* arcA

The *H. influenzae arcA* mutant exhibits increased expression relative to the parental strain of a set of genes (*lldD, lldP, fdxH, fdxI, fdhE, sucA, sucB* and *ndh*) encoding putative respiratory dehydrogenases, substrate transporters for these dehydrogenases, or enzymes involved in dehydrogenase assembly. These enzymes include α-ketoglutarate dehydrogenase, formate dehydrogenase and l-lactate dehydrogenase (LldD). Consistent with these results, LldD and a subunit of formate dehydrogenase (FdxG) have been detected as ArcA-regulated proteins in type b *H. influenzae* (De Souza-Hart *et al*., 2003). Substrates for these enzymes are created endogenously or available in the host. For example, l-lactate is produced at significant levels in the host with persistent levels in the blood ranging between 0 and 1.5 mM in resting, healthy individuals and levels as high as 4 mM in sepsis (Levraut *et al*., 1998). Expression levels of these enzymes correlate with their activity levels in *E. coli* (Iuchi and Lin, 1988), and this expression pattern is likely to signify increased cellular respiration in the mutant.

The observed anaerobic increase in the expression of a number of genes involved in energy metabolism in the *H. influenzae arcA* mutant is consistent with the reported role of ArcA in *E. coli* as a negative regulator of genes of the respiratory pathway and TCA cycle (Lynch and Lin, 1996a; Patschkowski *et al*., 2000; Oshima *et al*., 2002; Liu and De Wulf, 2004; Salmon *et al*., 2005). In addition, we detected ArcA regulation of *ndh*. The *E. coli ndh* encodes a non-proton-translocating NADH dehydrogenase (Matsushita *et al*., 1987). A second NADH dehydrogenase in *E. coli*, encoded by the *nuo* operon, is coupled to the generation of proton motive force (Weidner *et al*., 1993) and has no apparent homologue in *H. influenzae*. Although the *nuo* operon is repressed by ArcA in *E. coli* (Bongaerts *et al*., 1995), regulation of the *E. coli ndh* gene involves the oxygen-responsive regulator, FNR (Green and Guest, 1994; Meng *et al*., 1997). To our knowledge, transcriptional regulation of *ndh* by ArcA as detected in this study has not been reported previously. Together, these results demonstrate increased expression of multiple genes of cellular respiration in the ArcA mutant.

In the presence of oxygen, respiration generates endogenous ROI. *E. coli arcA* mutants exhibit increased rates of respiration (Nystrom *et al*., 1996; Vemuri *et al*., 2006). Potentially consistent with these results, a *S. enterica* serovar Enteritidis *arcA* mutant has been shown to be more susceptible to H_2_O_2_ compared with wild type (Lu *et al*., 2002). It is likely that increased levels of respiration are generated in the ArcA mutant, contributing to its hydrogen peroxide sensitivity.

### Positive control of oxidative stress resistance genes by ArcA

Deletion of *arcA* resulted in decreased expression of a set of genes with probable roles in oxidative stress resistance including *dps* (*HI1349),* encoding a putative homologue of ferritin-like iron storage proteins in other bacteria, and a putative operon containing genes similar to those mediating polyamine biosynthesis and transport in other organisms. A non-polar deletion of *dps* resulted in H_2_O_2_ sensitivity similar to that of the ArcA mutant under equivalent conditions, consistent with an important role for ArcA-mediated activation of this gene in H_2_O_2_ resistance.

The *dps* gene is conserved in all of the sequenced isolates of *H. influenzae* (Fleischmann *et al*., 1995; Harrison *et al*., 2005). Dps proteins bind iron and oxidize Fe(II) with H_2_O_2_ to form a stable ferric oxide mineral core within the cavity of the protein, thereby avoiding generation of toxic hydroxyl radicals mediated by Fenton chemistry (Zhao *et al*., 2002). Since the discovery that Dps in *E. coli* plays a role in protection against DNA damage from oxidative stress mediated by H_2_O_2_, homologues have been found to confer resistance to H_2_O_2_ in diverse bacteria (Chen and Helmann, 1995; Martinez and Kolter, 1997; Cooksley *et al*., 2003; Ishikawa *et al*., 2003; Ueshima *et al*., 2003; Halsey *et al*., 2004; Loprasert *et al*., 2004; Olsen *et al*., 2005; Pulliainen *et al*., 2005). HI1349 has approximately 18% identity and 20% similarity to *E. coli* Dps. Furthermore, HI1349 contains a conserved amino acid motif comprised of residues His38, His50, Glu54, Asp65 and Glu69 that are located at equivalent positions in the *Listera innocua* Dps and were implicated as sites for iron-binding based on structural analysis (Ilari *et al*., 2000). The Glu54 residue in the putative *H. influenzae* Dps represents a conserved variation of Asp at this equivalent position in other Dps proteins. Functional evidence of iron-binding capabilities of the corresponding residues has been obtained with the *Streptococcus suis* Dps protein (Pulliainen *et al*., 2005).

Our results indicate that the *H. influenzae dps*-like protein also functions in conferring resistance to H_2_O_2_, with the protection against transient exposure to H_2_O_2_ being more pronounced in anaerobically grown cells and lower in aerobically grown cells. The *dps* genes have been shown to be regulated by OxyR in the presence of exogenous H_2_O_2_ in multiple species including *E. coli* (Zheng *et al*., 2001), *Bacteroides* (Rocha *et al*., 2000), Archaea (Wiedenheft *et al*., 2005) and OxyR-dependant regulation of the *dps*-like gene of *H. influenzae* was recently observed (Harrison *et al*., 2006). In addition, *dps* mRNA and protein levels in *Borrelia burgdorferi* were higher in cultures grown microaerobically versus anaerobically, indicating redox regulation of this gene in this species (Seshu *et al*., 2004). To our knowledge, a role for ArcA in regulation of *dps* has not been demonstrated previously in any species. The role we have detected for ArcA in anaerobic expression of the putative *H. influenzae* Dps homologue, and the H_2_O_2_ sensitivity of the *dps* mutant versus the parental strain after anaerobic growth, provide evidence that a Dps protein can operate under a physiological condition not previously recognized to induce its upregulation. Because the *H. influenzae dps*-like gene was more critical for resistance to H_2_O_2_ in cells that were grown anaerobically versus aerobically prior to oxidant exposure, it is likely that cells grown under aerobic conditions rely more heavily on other systems including catalase, superoxide dismutase and peroxiredoxin-glutaredoxin (Bishai *et al*., 1994; D'Mello *et al*., 1997; Vergauwen *et al*., 2003), for resistance subsequent to exposure to H_2_O_2_. It is also likely that aerobic *dps* transcription, activated by OxyR in response to H_2_O_2_ (Harrison *et al*., 2006), and subsequent translation of sufficient amounts of Dps protein to afford protection require more time to confer protection than the transient exposure period used here. If cells are able to survive the initial exposure to H_2_O_2_, then Dps could accumulate, and we would predict that it could contribute to the multiple mechanisms of resistance to H_2_O_2_ present in *H. influenzae* growing aerobically.

In the Δ*arcA* mutant, we also observed decreased expression relative to the parent of a locus similar to the *speF-potE* operon of *E. coli* which encodes an inducible ornithine decarboxylase (*speF*), responsible for conversion of ornithine to putrescine, and the putrescine transport protein (*potE*) (Kashiwagi *et al*., 1991). Polyamines, such as putrescine and spermidine, have roles in a wide variety of biological processes and their optimal cellular concentrations are maintained by biosynthesis, degradation and transport (Igarashi and Kashiwagi, 1999). Polyamines have also been implicated in resistance to oxidative stress in *E. coli* and other organisms (Tkachenko *et al*., 2001; Chattopadhyay *et al*., 2003; Jung and Kim, 2003). The cellular amines putrescine, cadaverine and 1,3-diaminopropane are present in *H. influenzae* as measured by HPLC (Hamana and Nakata, 2000).

Downregulation of *speF* and *potE* in the *H. influenzae*Δ*arcA* mutant could disrupt the optimal balance of cellular polyamine levels, thereby contributing to oxidant sensitivity. However, a mutant containing a deletion of *speF*, *potE* and *HI0592* did not exhibit an appreciable increase in sensitivity to H_2_O_2_ exposure under the conditions tested. This result could potentially be attributed to the presence in *H. influenzae* of possible alternative pathways for production/uptake of polyamines. *HI0949* and *HI0946.1* have been shown to express the enzymatic activities required for 1,3-diaminopropane production (Ikai and Yamamoto, 1998). *H. influenzae potABCD* genes, whose *E. coli* counterparts function as a spermidine (preferential) and putrescine uptake system (Furuchi *et al*., 1991) could provide a similar capability to *H. influenzae*.

### ArcA, oxidative stress resistance and pathogenesis

Growth of *H. influenzae* within the human host likely requires adaptation to diverse conditions as the bacteria accumulate in biofilms on mucosal surfaces, invade the epithelium within or between cells, enter the bloodstream, or encounter oxidative defences of phagocytic cells. Decreased survival of the *arcA* mutant in the mouse model suggests that ArcA-mediated regulation is required during infection. Our results implicate ArcA in repression of genes of respiratory catabolism and activation of an oxidative stress resistance gene, *dps*, in addition to genes of polyamine metabolism. These regulatory effects of ArcA could protect cells growing under relatively low oxygen levels, a condition they likely encounter in venous blood or in biofilms on mucosal surfaces, against sudden oxidative stresses such as exposure to the oxidative defences of phagocytic cells. Consistent with this hypothesis, both *arcA* and *dps* exerted greater effects on hydrogen peroxide resistance in cells grown anaerobically than in cells grown aerobically prior to exposure. A pre-emptive role of certain oxidative stress resistance mechanisms has been proposed previously, such as anaerobic expression of an iron-dependant superoxide dismutase of *E. coli* (Kargalioglu and Imlay, 1994), but this aspect of resistance has received relatively little attention compared to defence mechanisms induced upon exposure to oxidants. Our results indicating *arcA*-dependant anaerobic activation of *dps* expression and the role of these genes in hydrogen peroxide resistance in *H. influenzae* demonstrate an additional mechanism by which bacteria can prepare for rapid transitions from low oxygen conditions to exposure to oxidative stress, conditions they likely encounter within the mammalian host.

## Experimental procedures

### Media and H. influenzae growth conditions

The non-encapsulated Rd derivative of *H. influenzae* type d (BA042), termed Rd in this report (Akerley *et al*., 2002) was grown at 35°C ± 1.5°C in Brain Heart Infusion supplemented with 10 μg ml^−1^ nicotinamide adenine dinucleotide and 10 μg ml^−1^ haemin (sBHI) on agar plates or in sBHI broth cultures shaken at 250 r.p.m. as indicated. DNA was transformed into naturally competent *H. influenzae* prepared as previously described (Barcak *et al*., 1991). Kanamycin (Km), gentamicin (Gm) and tetracycline (Tet) were added to sBHI at 20 μg ml^−1^, 10 μg ml^−1^ and 8 μg ml^−1^ respectively. *H. influenzae* cultures grown in unaerated culture containers filled and sealed to exclude air to approximate anaerobiosis were termed ‘anaerobic’ for simplicity. *H. influenzae* is a haem auxotroph and requires haem for aerobic growth, yet grows to equivalent levels in these sealed containers in the presence and absence of exogenous haem, suggesting oxygen levels are very low or absent in sealed tubes (data not shown). Aerobically grown *H. influenzae* are cultured in 10 ml of sBHI in 500 ml Erlenmeyer flasks. The generation times for parent strain (RdV), *arcA* deletion mutant (RAA6V) and the complemented strain (RAA6C) were 29 ± 3 min (*n* = 3), 31 ± 1.6 min (*n* = 3), and 31 ± 5 min (*n* = 3), respectively, for aerobic growth; 67 ± 3 min (*n* = 3), 71 ± 8 min (*n* = 3), and 69 ± 3 min (*n* = 3), respectively, for anaerobic growth.

### *Plasmid and* H. influenzae *strain construction*

Standard molecular biology methods were used for plasmid construction and Northern blot analysis (Ausubel *et al*., 1995). Strain RdV was generated by transforming Rd with vector pXT10 and selection with tetracycline for homologous recombination at the *xyl* locus (Wong and Akerley, 2003). Strain RAA6 contains a non-polar, in-frame deletion of the *arcA* protein coding sequences (Georgellis *et al*., 2001b). Vector pXT10 was introduced into RAA6 at the *xyl* locus to generate RAA6V. RAA6C contains the deletion of *arcA* with a wild-type copy of *arcA* introduced *in trans* at the *xyl* locus as described previously (Georgellis *et al*., 2001b). Strains Rcp5 and Rcp19 containing transposon insertion mutations in *lpsA* and *galU,* respectively, were generated by *in vitro* transposon mutagenesis with the *Himar1* derivative *magellan1* as previously described (Akerley *et al*., 2002), and mapped by DNA sequence analysis of PCR-amplified transposon junctions. Rcp5 and Rcp19 contain *magellan1* insertions in their coding sequences at nucleotide positions 635 in *lpsA* and 264 of *galU* respectively. *H. influenzae* Rd carrying *lacZ* was made by cloning a promoterless *E. coli lacZ* into the SapI sites of pXT10 and introduced into Rd at the *xyl* locus.

A non-polar, in-frame deletion of *HI1349* (*dps*) was created by replacement of the protein coding sequences with the *aacC1* gentamicin resistance cassette to create Rdps by PCR ‘stitching’ as follows: A 1172 bp PCR product containing the 5′ flanking region of *HI1349* was amplified from Rd with primers 1350-3 (5′-TTACAAAGAATAATACTCTAATTCTAC) and 1349-5outgent (5′-ATTCGAGAATTGACGCGTAATAATTTCCTTTTTCTAGTTGAA). A 1881 bp PCR product containing the 3′ flanking region of *HI1349* was amplified from Rd with primers 1349-3outgent3 (5′-GTTCAAGCCGAGATCTGAATAAATTTCAACGCTAACGAA) and 1348-5upout (5′-TCAAGATGTTTTCTATTTTTCTCG). A 791 bp fragment containing the *aacC1* gentamicin resistance cassette was amplified with primers gentMluI5 (5′-ACGCGTCAATTCTCGAATTGACAT) and gent-3′ (5′-GATCTCGGCTTGAACGAATTGTTA) from pBSL182 (Alexeyev and Shokolenko, 1995). The 1172 bp, 1881 bp and 791 bp products were stitched in a PCR reaction with primers 1350-3 and 1348-5upout. The resultant 3.84 kb product was introduced into Rd and Gm^R^ transformants were selected on sBHI agar containing Gm to create strain Rdps.

To generate RdpsV, the vector pXT10 was introduced into the *xyl* locus of Rdps. RdpsC was created as follows: A 333 bp fragment containing the putative *hel* (*HI0693*) promoter was PCR-amplified with primers 692–5ATGoutSap2 (5′-AACTGCAGATCTGCTCTTCAATGCATTTGAAACATATCCCAAGT) and hel5′ATGout2 (5′-CAGGGTATAGTAAGTCTTTCTGA) from Rd. A 525 bp product containing the *HI1349* gene was amplified from Rd with primers 1349SDATG (5′-ACTTACTATACCCTGTAGAAAAAGGAAATTATTATGTCA) and 1349-3Sap (AAAGATCTGCAGGCTCTTCTTTAATTATGGCAAGTTTGGCAAGC). These two products were stitched in a PCR reaction with primers 692-5ATGoutSap2 and 1349-3Sap. The resultant 858 bp PCR product was digested with SapI and cloned into the SapI sites of pXT10. This plasmid was introduced into the *xyl* locus of strain Rdps and Tet^R^ transformants were selected on sBHI agar containing Tet to create strain RdpsC.

A non-polar, in-frame deletion of *HI0592*, *speF* and *potE* was created as follows: A 2086 bp PCR product containing the 5′ flanking region of *HI0592* was amplified from Rd with primers 594 5′ORF (5′ATGGATGCATCCAAAAAG) and 592out + kan (5′-TTGAATATGGCTCATAGGAAAAATCCCTCTTTCTATCTA). A 1054 bp PCR product containing the 3′ flanking region of *HI0590* was amplified from Rd with primers potE 3 + kan (5′-GATGAGTTTTTCTAAAAAAGAAACGCCTACATCTTAATG) and 588–5int (5′-AAAGAGGATTATTAATTGAGTTAC). A 818 bp fragment containing the kanamycin resistance gene, *aphI* from Tn*903* was PCR-amplified with primers kan5 + ATG (5′-ATGAGCCATATTCAACGGGAAAC) and kan3′ + TAA (5′-TTAGAAAAACTCATCGAGCATCAAA). The three resultant products were used as template in PCR to amplify a 3921 bp ‘stitched’ product with primers 594-5′ORF and 588–5int. The 3921 bp product was introduced into RdV and Km^R^ transformants were selected on sBHI agar containing Km to create strain RputV. All transformants were verified by PCR analysis.

### Murine bacteraemia model

*Haemophilus influenzae* were grown to logarithmic phase (OD_600_ = 0.3) as 20 ml of cultures in 50 ml shake flasks at 35°C. Five- to six-week-old C57BL6 mice were inoculated by the IP route at a dose of 2–8 × 10^8^ cfu. Blood (5 μl) was collected daily by tail vein sampling and serially diluted in BHI for cfu determination and results compared via the one-way anova with the Bonferroni correction. Experiments were conducted with approval and in accordance with guidelines of the University of Massachusetts Institutional Animal Use and Care Committee.

### Serum bacteriocidal assays

Serum bactericidal testing was performed as described previously (McQuillen *et al*., 1994). Briefly ∼2000 cfu of bacteria grown anaerobically to the mid-log phase were incubated with or without normal human serum (NHS) (concentration of NHS specified for each experiment) in a final reaction mixture volume of 150 μl. Aliquots of NHS treated versus untreated samples were plated on sBHI agar plates at 0 and 30 min. In all cases, the number of bacteria recovered from treated and untreated samples at 0 min were equivalent. Survival was calculated as the ratio of the number of cfu recovered at 30 min relative to cfu recovered from untreated samples. An initial study with a range of serum concentrations (1, 2, 3, 4, 5 and 10% NHS) revealed that both Rd and RAA6 were affected similarly at each dose with 4% NHS or above, killing greater than 99% of cells of either strain and both strains had an approximate IC50 of 2%. Therefore, three replicate experiments were conducted with 1% and 2% NHS as indicated in the text.

### Northern and RT-qPCR

Total RNA from *H. influenzae* Rd was obtained from cultures grown anaerobically in sBHI to OD_600_ = 0.3–0.4 as described above. RNA was isolated using TRIzol Reagent (Invitrogen), treated with DNase I (Ambion) and phenol extracted. For Northern blotting, 6 μg of total RNA was separated by electrophoresis on a 1.5% agarose gel containing 1.1% formaldehyde and transferred to a Nytran nylon membrane (Amersham Pharmacia Biotech). Probes were generated by amplification from Rd using 5′ and 3′ primer pairs for *ndh* (*HI0747*) (5′-ATGAAAAACGTCGTGATC and 5′-ATGCAATTTTAATCTTGGTTTTAAATAAC), *lldD* (*HI1739.1*) (5′-ATGATTATTTCATCAGCTAG and 5′-AAGTTTACTTAGATCAACC), *fdxH* (*HI0007*) (5′-ATGGCTGGAACTGCTCAAGGCG and 5′-GAAACACGATCTACACAAAGAG), *fdxI* (*HI0008*) (5′-ATGAGTAAAATTGAAATTAGCAAC and 5′-AGATACCAGTGAATAACATAAAAG), *fdhE* (*HI0009*) (5′-ATGAGTATCAAAATCTTATC and 5′-TGCTTCTTCTGCAGGAAAAATAAATG), *lldP* (*HI1218*) (5′-ATGCTGTCTTTTATTCTAAG and 5′-TAGATTATAAAATAAAGGTAC), *sucB* (*HI1661*) (5′-ATGGCAATCGAAATTCTTG and 5′-GATTTCTAATAACAATCTTG), *HI0592* (5′-ATGCTATTTCGTACATATATAC and 5′-GAGAGCCCTGTTGGATG), *potE* (*HI0590*) (5′-ATGAGTGCTAAAAGCAATAAAATTG and 5′-TTTTTTAAGATCAAATTTGTAAG) and *HI1349* (5′-AACTGCAGATCTGCTCTTCAATGTCAAAAACATCAATCGGACTA and 5′-AAAGATCTGCAGGCTCTTCTTTAATTATGGCAAGTTTGGCAAGC). PCR products were labelled with the Gene Images AlkPhos Direct Labeling Kit and signals visualized with CDP-*Star* chemiluminescent detection system (Amersham Pharmacia Biotech). The *lldD* Northern blot was performed by stripping of the *lldP* probe from the membrane followed by hybridization with the *lldD* probe. Washing and hybridization conditions were according to the manufacturer's instructions.

Quantification of mRNA expression of the *HI1349* (*dps*) and *HI0802* (*rpoA*) from strains RdV, RAA6V and RAA6C with RNA samples from four independent cultures used in the microarray analysis was performed using iQ SYBR Green Supermix (Bio-Rad Laboratories) in quantitative real-time PCR measured with the DNA Engine Opticon II System (MJ Research). Briefly, 2.5 μg of DNase I-treated total RNA from the above strains was used as template in cDNA synthesis using random primers (New England Biolabs) and SuperScript II reverse transcriptase (Invitrogen). One-tenth of the reverse transcriptase reactions was used as template in qPCR for amplification using 5′ and 3′ primer pairs for *dps* (5′-AACTGCAGATCTGCTCTTCAATGTCAAAAACATCAATCGGACTA and 5′-ACATTCTTGTGCCTCACTTACTGC) and *rpoA* (5′-GTAGAAATTGATGGCGTATTG and 5′-TCACCATCATAGGTAATGTCC). Real-time cycler conditions were as follows: 95°C for 3 min, followed by 39 cycles of 96°C for 20 s, 58°C for 30 s and 72°C for 30 s, followed by one cycle of 72°C for 7 min. Fluorescence was read at 72, 74, 76 and 78°C and normalized to the housekeeping gene, *rpoA,* which encodes the alpha subunit of RNA polymerase. Control reactions were performed in parallel with mock cDNA reactions generated without reverse transcriptase to verify specific amplification. Product sizes were confirmed by agarose gel electrophoresis.

### Microarray analysis

Total RNA from four independent cultures of *H. influenzae* Rd grown anaerobically to OD_600_ = 0.3–0.5 was obtained and treated with DNase I as described above. Thirty micrograms of RNA from each quadruplicate culture was used as template for generation of probes by reverse-transcription in the presence of random primers, essentially as described previously (Wong and Akerley, 2005) except that biotinylated nucleotides (Mergen, San Leandro, CA) were used in the reverse-transcription. Biotinylated cDNAs were hybridized to glass slide microarrays containing 45-mer oligonucleotides representing a total of 1697 genes of the *H. influenzae* KW20 genome in addition to negative controls specific for human genes. Array printing, fluorescent labelling, hybridization to microarrays, and array scanning was conducted by Mergen (San Leandro, CA). The total signal intensity for every gene represented on the array was corrected by subtracting the local background and normalized by dividing by the mean of the values for all of the genes represented on the array. Bacterial cultures, RNA isolation, labelling and hybridizations were conducted independently to obtain four experimental replicates for each strain. The corrected signal intensity for each gene represents the mean of separate hybridization experiments conducted with labelled cDNAs from each of the four independent cultures of each strain. Expression ratio data were generated by comparing the corrected mean signal intensity values from arrays hybridized to cDNAs generated from the parent strain versus the *arcA* mutant or complemented *arcA* strain versus the *arcA* mutant. Statistical analysis of the expression data were performed using the Cyber-T Bayesian statistics framework (Baldi and Long, 2001) available as a web interface from the Institute for Genomics and Bioinformatics at the University of California, Irvine (http://visitor.ics.uci.edu/genex/cybert). Genes whose expression ratios had Bayesian *P*-values based on the regularized *t*-test ≤ 0.0001 and showed ≥ 2.0-fold differential gene expression levels were considered to exhibit significant differences. Mean signal intensities greater than or equal to twofold above the mean local background averaged over all the spots on all of the arrays were considered to have reached the threshold value for significant gene expression.

### Hydrogen peroxide sensitivity assays

Survival after exposure to H_2_O_2_ was evaluated for anaerobically or aerobically grown *H. influenzae*. Aerobically grown *H. influenzae* was prepared by diluting overnight cultures 1:200 in sBHI in standard culture tubes and growing at 35°C with shaking at 250 r.p.m. to an OD_600_ = 0.1–0.2. Triplicate cultures of each strain were inoculated at 0.005 OD_600_ units ml^−1^ into 10 ml of sBHI in 500 ml Erlenmeyer flasks and grown at 35°C with shaking at 250 r.p.m. to an OD_600_ = 0.3–0.4. Anaerobic cultures were prepared by diluting overnight standing cultures to 0.005 OD_600_ units ml^−1^ in sBHI (triplicates per strain) in glass vials filled to exclude air and grown at 35°C with shaking at 250 r.p.m. to an OD_600_ = 0.3–0.4. Cells from 1 ml of each culture were pelleted and resuspended in 1 ml of MIc medium (Barcak *et al*., 1991). The H_2_O_2_ sensitivity assay was conducted in Costar 24-well cell culture plates seeded with 100 μl per well. An equal volume of MIc medium with and without 1 mM H_2_O_2_ was added (final concentration of 0.5 mM) and the plate was shaken at 250 r.p.m. at 35°C for 10 min followed by addition of 10 mM sodium pyruvate to quench H_2_O_2_ (Maciver and Hansen, 1996) to all samples. H_2_O_2_ treated and untreated cells were diluted and plated onto sBHI agar plates for cfu determination. Differences were compared via the one-way anova with Bonferroni post-tests.

## Supplementary material

The following supplementary material is available for this article:

Table S1 Microarray data of parent vs. Δ*arcA* mutant in *H. influenzae Rd*. Microarray data was analyzed using the Cyber T statistics program. Input for the Cyber T program is the corrected signal intensity values from 1697 genes representing the genome of *H. influenzae* and control probes (see Col. 1 definitions in table) hybridized with cDNA from quadruplicate cultures of the RdV parental strain, Δ*arcA* mutant, RAA6V, and complemented strain, RAA6C grown anaerobically. In this table, the Cyber T analysis represents the comparison between the parent strain and Δ*arcA* mutant. The column heading definitions are listed at the end of the data file.

Table S2 Microarray data of arcA complemented strain vs. Δ*arcA* mutant in *H. influenzae* Rd. Microarray data was analyzed using the Cyber T statistics program. Input for the Cyber T program is the corrected signal intensity values from 1697 genes representing the genome of *H. influenzae* and control probes (see Col. 1 definitions in table) hybridized with cDNA from quadruplicate cultures of the RdV parental strain, Δ*arcA* mutant, RAA6V, and complemented strain, RAA6C grown anaerobically. In this table, the Cyber T analysis represents the comparison between the complemented strain and Δ*arcA* mutant. The column heading definitions are listed at the end of the data file.

Table S3 Comparsion of *arcA* microarray expression data between *H. influenzae* and *E. coli*. Similarities and differences across species in putative ArcA regulons. *H. influenzae arcA* microarray expression data (from Tables 1 and 2, columns RAA6V/RdV) was compared to *E. coli arcA* microarray expression data of Salmon *et al*. (Salmon, K.A., *et al*. 2005. J. Biol. Chem. 280: 15084–15096) and Liu and DeWulf (Liu, X. and P. De Wulf. 2004. J. Biol. Chem. 279: 12588–12597). Putative *E. coli* homologs in bold have expression data from either microarray sets of Salmon *et al*. or Liu and De Wulf that show agreement with the *H. influenzae* array data in anaerobic expression ratios in the Δ*arcA* mutant, RAA6V vs. the parent strain, RdV. Fold change values represent an increase (positive) or decrease (negative) in gene expression in the *ΔarcA* mutant vs. parent strain, RdV. *E. coli* homologs with blank entries are genes whose expression levels did not meet the authors′ criteria for minimum signal detection on the array.

This material is available as part of the online article from: http://www.blackwell-synergy.com/doi/abs/10.1111/j.1365-2958.2007.05747.x

(This link will take you to the article abstract).
