# Supplementary material for: The ArcA regulon and oxidative stress resistance in Haemophilus influenzae
Source: Mol Microbiol. 2007 Jun 1;64(5):1375–90. doi: 10.1111/j.1365-2958.2007.05747.x (PMC1974803; doi:10.1111/j.1365-2958.2007.05747.x)
Supplement: Table S3 — Comparsion of arcA microarray expression data between H. influenzae and E. coli. Similarities and differences across species in putative ArcA regulons. H. influenzae arcA microarray expression data (from Tables 1 and 2, columns RAA6V/RdV) was compared to E. coli arcA microarray expression data of Salmon et al. (Salmon, K.A., et al. 2005. J. Biol. Chem. 280: 15084–15096) and Liu and DeWulf (Liu, X. and P. De Wulf. 2004. J. Biol. Chem. 279: 12588–12597). Putative E. coli homologs in bold have expression data from either microarray sets of Salmon et al. or Liu and De Wulf that show agreement with the H. influenzae array data in anaerobic expression ratios in the ΔarcA mutant, RAA6V vs. the parent strain, RdV. Fold change values represent an increase (positive) or decrease (negative) in gene expression in the ΔarcA mutant vs. parent strain, RdV. E. coli homologs with blank entries are genes whose expression levels did not meet the authors′ criteria for minimum signal detection on the array. [file mmi0064-1375-ts3.pdf]

Table 3S. Comparsion of *arcA* microarray expression data between *H. influenzae* and *E. coli*

H. influenzae		RAA6V/ RdV			Salmon *et al.* *arcA-/ arcA +*		Liu and De Wulf *arcA-/ arcA +*	
Gene ID	Function	Fold change	*P* value	*E. coli* homolog	Fold change	*P* value	Fold change	*P* value
HI0007	Formate dehydrogenase, beta subunit (*fdxH*)	7.8	1.12E-09	*fdnH*	-1.62	3.26E-02		
				fdoH	**2.32**	**5.08E-02**		
HI0008	Formate dehydrogenase, gamma subunit (*fdxI*)	9.7	5.42E-09	*fdnI*	-2.18	5.89E-03		
HI0009	FdhE protein (*fdhE*)	9.7	8.39E-10	***fdhE***	**1.92**	**1.65E-02**		
HI0018	Uracil DNA glycosylase (*ung*)	2.5	1.01E-05	*ung*				
HI0026	Lipoate biosynthesis protein A (*lipA*)	2.6	4.08E-07	***lipA***	**1.82**	**1.47E-01**		
HI0174	Conserved hypothetical protein	2.3	1.75E-05	*ycfB*	-1.36	9.44E-02		
HI0608	Conserved hypothetical protein	5.5	3.65E-10	*yfbS*	-1.91	4.08E-03		
HI0747	NADH dehydrogenase (*ndh*)	10.7	4.06E-11	***ndh***	**3.93**	**6.47E-03**	-3.5	2.64E-02
HI0889	Serine hydroxymethyltransferase (serine methylase) (*glyA*)	2.4	4.11E-06	*glyA*	1.04	8.47E-01		
HI0890	Dephospho-CoA kinase	2.5	4.83E-05	*coaE (yacE)*				
HI1218	L-lactate permease (*lldP*)	44.0	1.20E-08	*lctP (lldP)*			**11.4**	**2.0E-04**
HI1444	5,10 methylenetetrahydrofolate reductase (*metF*)	5.6	4.91E-06	*metF*				
HI1661	2-oxoglutarate dehydrogenase E2 component (*sucB*)	2.8	5.33E-07	***sucB***	**15.50**	**1.14E-03**		
HI1662	2-oxoglutarate dehydrogenase E1 component (*sucA*)	2.6	6.71E-07	***sucA***	**11.14**	**1.85E-03**		
HI1727	Argininosuccinate synthetase (*argG*)	4.8	5.02E-07	*argG*	-1.82	5.94E-03		
HI1728	Conserved hypothetical protein	3.5	1.54E-06	*yfeP*	-1.78	4.54E-03		
HI1730	Conserved hypothetical protein	3.0	1.48E-06	*ybgK*				
HI1731	Conserved hypothetical protein	5.9	2.52E-09	*ybgJ*				
HI1739.1	L-lactate dehydrogenase (*lldD*)	3.2	1.79E-10	*lctD (lldD)*			**11.4**	**2.0E-04**
HI0590	Putrescine-ornithine antiporter (*potE*)	-2.5	3.06E-08	***potE***	**-1.50**	**1.87E-02**		
HI0591	Ornithine decarboxylase (*speF*)	-4.0	2.54E-08	*speF*				
HI0592	Conserved hypothetical protein	-4.4	4.62E-08	no homolog				
HI0884	Aerobic respiration control protein ArcA (*arcA*)	-654.8	2.72E-12	***arcA***	**-2.09**	**2.37E-03**		
HI1349	Conserved hypothetical protein, similar to dps protein family	-3.4	1.43E-07	*dps*				

Similarities and differences across species in putative ArcA regulons. *H. influenzae arcA* microarray expression data (from Tables 1 and 2, columns RAA6V/ RdV) was compared *to E. coli arcA* microarray expression data of Salmon et al. (Salmon, K.A., et al. 2005. J. Biol. Chem. 280: 15084-15096) and Liu and De Wulf (Liu, X. and P. De Wulf. 2004. J. Biol. Chem. 279:12588-12597).
Putative *E. coli* homologs in bold have expression data from either microarray sets of Salmon *et al* . or Liu and De Wulf that show agreement with the *H. influenzae* array data in anaerobic expression ratios in the Δ*arcA* mutant, RAA6V vs. the parent strain, RdV. Fold change values represent an increase (positive) or decrease (negative) in gene expression in the Δ*arcA* mutant vs. parent strain, RdV. *E. coli* homologs with blank entries are genes whose expression levels did not meet the authors' criteria for minimum signal detection on the array.
